# Water ascent in trees and lianas: the cohesion-tension theory revisited in the wake of Otto Renner

**DOI:** 10.1007/s00709-016-1009-4

**Published:** 2016-08-04

**Authors:** Friedrich-Wilhelm Bentrup

**Affiliations:** Abteilung Pflanzenphysiologie, Universität Salzburg, Hellbrunnerstr. 34, 5020 Salzburg, Austria

**Keywords:** Cohesion-tension theory, Water ascent, Multi-force theory, Xylem pressure, Emboly repair, Water status online monitoring, Otto Renner, Max Planck

## Abstract

The cohesion-tension theory of water ascent (C-T) has been challenged over the past decades by a large body of experimental evidence obtained by means of several minimum or non-invasive techniques. The evidence strongly suggests that land plants acquire water through interplay of several mechanisms covered by the multi-force theory of (U. Zimmermann et al. New Phytologist 162: 575–615, 2004). The diversity of mechanisms includes, for instance, water acquisition by inverse transpiration and thermodynamically uphill transmembrane water secretion by cation-chloride cotransporters (L.H. Wegner, Progress in Botany 76:109–141, 2014). This whole plant perspective was opened by Otto Renner at the beginning of the last century who supported experimentally the strictly xylem-bound C-T mechanism, yet anticipated that the water ascent involves both the xylem conduit and parenchyma tissues. The survey also illustrates the known paradigm that new techniques generate new insights, as well as a paradigm experienced by Max Planck that a new scientific idea is not welcomed by the community instantly.

## Introduction

The cohesion-tension theory (C-T theory) by Boehm (1893) and Dixon and Joly (1894) postulates that the water ascent in trees is exclusively due to the transpirational pull from continuous water columns in the xylem conduit running from the roots to the leafs. This pull is argued to create tension gradients of several MPa in order to overcome the gravitational force and frictional resistances. Water under tension (negative pressure) is in a metastable state. By means of his ingenious vacuum line-based experiment on leafy twigs, Otto Renner (1911) demonstrated that the xylem water indeed may exist under tension. Therefore, he favored the C-T theory, but nevertheless argued “that the turgor of the xylem parenchyma cells attains equilibrium with the pressure of the adjacent vessels.” Clearly, hydraulic coupling of these tissues would preclude xylem tensions of several MPa inferred by the C-T theory. In fact, Renner (1925) found moderate tensions yielding a mean value of 0.32 ± 0.2 MPa (*n* = 43). Textbooks did not present these data, rather introduced the pressure bomb technique of Scholander et al. (1965) to support the C-T theory. The output of this tool, the *balancing pressure*, is measured on excised leafy twigs, and is considered to reflect 1:1 the xylem tension in the intact plant.

This survey illustrates how recently developed minimum invasive or non-invasive techniques challenge this historical liaison of C-T theory and Scholander bomb. Water acquisition of trees and lianas indeed relies upon an amazing diversity of mechanisms, rather than exclusively on hydrostatic pressure gradients in the xylem conduit postulated by the C-T theory. The multi-force theory of water acquisition proposed by U. Zimmermann et al. (2004) provides a powerful concept to elucidate the plant water status. Meanwhile, this concept includes the molecular biology of active transmembrane water transport as well as telemetric online-monitoring of the plant water status in the field.

## New techniques generate new insights

Progress of cell biology over the past decades conspicuously depended on the development of non-invasive or minimum invasive techniques, for instance fluorescent probes, laser, and ^1^H-NMR microscopy, and of cell pressure probes to elucidate the plant cell water status, i.e., a turgor pressure probe (Zimmermann et al. 1969) and a xylem pressure probe (Balling et al. 1988). Direct continuous recording of the tension (negative pressure) in the xylem conduit by means of the xylem pressure probe challenged the popular pressure bomb technique (Scholander et al. 1965). Hence, several laboratories compared both techniques. Balling and Zimmermann (1990) showed that the Scholander bomb did not read the actual tension in the xylem vessels of *Nicotiana* plants. A joint study by several laboratories on maize and sugarcane showed that the balancing pressure reflects the xylem pressure on non-transpiring plants only (Melcher et al. 1998).

On a tropical liana, *Tetrastigma voinierianum*, filling a greenhouse up to a height of 10 m, the xylem pressure probe recorded transpiration-driven diurnal changes of the xylem tension never exceeding 0.4 MPa (Benkert et al. 1995; Thürmer et al. 1999). For instance, at noon, the peak xylem tension was 0.4 MPa (absolute pressure −0.4 MPa), and the turgor pressure had dropped from 0.45 to 0.05 MPa. The osmolarity of the parenchyma cells was 0.82 ± 0.06 MPa (*n* = 101). These data clearly indicate the 1:1 hydraulic coupling between xylem parenchyma and vessels predicted by Otto Renner (1911). Concomitant bomb experiments, however, yielded inconsistent values between 0.1 and 1 MPa. The paper by Benkert et al. (1995) was accepted after 8 months and eight reviews. One reviewer noted: “my graduate students can do better Scholander experiments than these authors” and concluded “So if the cohesion theory is out, what according to the authors can account for xylem water flow? What they suggest is physical nonsense or voodoo.”

We had argued that our data are not consistent with the C-T theory. Moreover, we had introduced the thermodynamics of irreversible processes proposed by Plumb and Bridgman (1972). These authors had pointed out that any water flow is driven by gradients in the chemical activity of water, and argued that the high tensions inferred by the C-T theory were not necessary at all, indeed had not been measured so far. A gradient of gel-like filamentous structures attached to the walls of the xylem conduit, for instance, would lift the water column even without a hydrostatic pressure gradient; these authors also suspected that the high pressure Scholander et al. (1965) had needed to squeeze water out of mangrove twigs could be due to gels in the xylem water rather than due to ultrafiltration by the plasma membrane (i.e., by inverse osmosis). Indeed, this suspicion later was confirmed (see below). Their paper evoked a letter to the editor of *Science* filed by five prominent proponents of the C-T theory, who for different reasons did not comply with this critique (Scholander et al. 1973).

### ^1^H-NMR microscopy: nuclear magnetic resonance imaging of water

This non-invasive technique lends itself to obtain both the microscopic location and time-resolved movement of water within the intact plant, because the magnetic spin signature of the proton in H_2_O differs, whether the water molecule is mobile in the xylem or bound, for instance, to the cell wall. NMR imaging of the intact plant were established by the laboratories of Axel Haase and Ulrich Zimmermann (Rokitta et al. 1999).

By means of flow-sensitive NMR imaging as well as application of the turgor and xylem pressure probes to a 14-m-long specimen of the liana *Epipremnum aureum* in a vertical, horizontal, and inverse position, respectively, Wistuba et al. (2000) recorded diurnal changes and driving forces of xylem water flow caused by root to apex gradients of xylem tension and osmotically based turgor pressure gradients. (Note that the gravitational force is ineffective in the horizontal position.) The turgor pressure probe revealed a longitudinal osmotic pressure gradient. Hence, hydraulic coupling of xylem and parenchyma tissues apparently warrants a transpiration-independent anti-gravitational water flux from the root to the apex.

## Water ascent in plants: do ongoing controversies have a sound basis

Under this heading, a perspectives article of Wei et al. (1999b) drew attention to a paper by Wei et al. (1999a) comparing pressure bomb and xylem pressure probe measurements. This original paper displays a surprising number of substantial flaws of theory, experimentation, and documentation, and led us to an elaborate reply (Zimmermann et al. 2000). In particular, with reference to Plumb and Bridgman (1972), we pointed out that electric circuit diagrams do not provide a sound basis for the analysis of plant water relations. Above all, we noted that a sound basis for the elucidation of the water ascent would call for experiments on trees rather than on maize plants.

Nevertheless, the senior author of this maize study extended his audience by a *concepts* article nicely illustrated by a coast redwood, *Sequoia sempervirens* (Tyree 2003). The paper Wei et al. (1999a) is cited only briefly: “…measurement of negative pressure in xylem made with a cell pressure probe 5-15 years ago failed to confirm the C-T theory. But an improved pressure probe technique has now proved that the mechanism functions as supposed. How do plants do it and what other limitations on plant performance result?”

In fact, the “cell pressure probe” is the xylem pressure probe of Balling et al. (1988). Wei et al. pretend to have improved it by filling the glass capillary with oil instead of water. However, since oil is compressible, this change does not improve the bomb technique (see Melcher et al. 1998).

Incidentally, Tyree (2003) tuned the *Nature* reader to the topic by a subtitle: *Plant hydraulics. When you*’*re a large organism and made of wood*, *you can*’*t have a heart or other contractile organs*, *but you still need to move fluids to live. How is this done*? This subtitle raised a comment from Tanner (2004) who pointed out that the comparison of the heart-driven blood flow with the transpiration-driven water flow is misleading, because the latter is inevitably coupled to photosynthesis, but not essential to keep the plant alive.

Koch et al. (2004) reported on the classical experiment of recording height-dependent profiles of xylem tension by means of the pressure bomb. From five coast redwood specimen including the tallest tree on earth (112.7 m), twigs were collected between 50 and 110 m height. They report a linear increase of the balance pressure which, however, does not meet the slope of a vertical water column (0.01 MPa m^−1^). Yet this result is argued to support the C-T theory. Unfortunately, the methods section of this paper lacks crucial details, i.e., the time interval between twig collection and measurement, as well as corresponding height profiles of both temperature and relative humidity. The formidable task of twig collection from such high trees is only described by “Access to the treetop was achieved by arborist-style techniques.” For the significance of these experimental details, see below. This paper was welcomed by Ian Woodward (2004) who pointed out that a 112.7-m-high redwood resembles a 30-storey building, and summarized the paper to “indicate that fundamental control of maximum tree height is water supply to treetop…. They found that the maximum tension is close to the point of embolism, establishing this value as the first major control on height.” But he also notes that “flow through the xylem is slow; water entering the base of a redwood trunk could take as long as 24 days to reach the tree top.” Hence, tall trees plausibly might capture fog coming from the sea. Indeed, water uptake by the canopy of trees is quite real (see below).

## A Tansley Review shakes the cohesion-tension theory

In 2004, a Tansley Review was published by four scientists from Würzburg (Zimmermann et al. 2004). Based upon more than 300 references, plant water relations were subjected to a careful scrutiny of the techniques designed to support the C-T theory; cf. APPENDIX 2 on “Xylem vulnerability, imaginary xylem pressures, and other hypotheses.” The immense body of experimental evidence led the authors to propose a multi-force theory of water ascent in trees, that is, an interplay of several forces including cohesion, tension, capillarity, cell osmotic pressure gradients, xylem-phloem re-circulation, and hydrogel-bound gradients of the chemical activity of water. This “watergate model” clearly implies a functional segmentation of the xylem conduit. The conclusions chapter start as follows: “When reading the voluminous old literature, we were amazed at the profound insights of the scientists at that time into the diversity of mechanisms that might be involved in water ascent against gravity. The question is why this and more recent work challenging the view that tension is the only driving force for water lifting was ignored in the last 4 decades by many plant physiologists.” The authors summarize by stating “that the arguments of the proponents of the Cohesion Tension Theory are completely misleading.”

In a Letter to the Editor, 45 protagonists of the C-T theory responded to this review (Angeles et al. 2004): “We, the undersigned, believe that this review is misleading in its discussion of the many recent papers which demonstrate that the fundamentals of the Cohesion-Tension theory remain valid”. They close noting “We wish the readers of New Phytologist to know that the Cohesion –Tension Theory is widely supported as the only theory consistent with the preponderance of data on water transport in plants.”

These authors also sent a complaint to the Editor-in-Chief Ian Woodward. The caliber of this complaint raised a reply by Robert Hamilton, President of the Ecological Society of America: “While I am not a proponent of Zimmerman’s views, I must say that I am greatly disappointed in a scientific community that advocates the silencing of an established and deputable journal like New Phytologist because the journal publishes something that goes against mainstream science…there is no reason for the mainstream of science to make any sort of effort to silence Zimmerman…Too bad the era where major players feel the need to silence critics in science is not well behind us…

What are the supposedly ignored fundamentals of the C-T theory? The reader might prefer to ask for convincing experimental evidence to prove this 100-year-old theory; that is, evidence for continuous water columns reaching from the root to canopy of tall trees.

In fact, Westhoff et al. (2009a) presented evidence from 17- to 25-m-tall birch trees for discontinuous water columns. On trunk and branch pieces of 51 felled trees, the filling status of the vessels had been measured all year round by a broad array of techniques, including NMR imaging of mobile/cohesive water in the xylem, infusion experiments using dye, ^86^Rb^+^ or D_2_O, respectively, and xylem pressure measurements on intact trees and branches. A coherent body of data revealed that mobile/cohesive water preferentially exists at intermediate heights of the trunk, but clearly less at the base or top of tree. Upper branches contained more mobile/cohesive water than lower branches. Thus, water lifting apparently involves short-distance tension gradients and water mobilized from parenchyma tissues and the heartwood, as well as moisture uptake through lenticels.

Since a functional segmentation of the xylem conduit is not consistent with the C-T theory, publication of this paper was tenacious; one reviewer grumbled… “two sentences essentially say that the pressure bomb is misleading… how do these authors explain the classical patterns of leaf moisture release, which Tyree and Hammel (1972) analysed many years ago, and which are now a normal part of most plant physiology texts?”

## Water acquisition by pectin-like wall deposits

The historical encounter of the Scholander bomb and the mangrove eventually revealed that plants may acquire water by binding to mucilaginous (pectic) compounds in the xylem water, rather than by ultrafiltration of sea water (Scholander et al. 1965; Zimmermann et al. 1994). This mechanism enables trees to draw water from high-salinity substrates (Zimmermann et al. 2002). As outlined above, Plumb and Bridgman (1972) had suggested already that instead of a hydrostatic pressure gradient to overcome the gravitational force, water activity gradients due to fibrillar cell wall deposits would suffice for water lifting. This mechanism has been nicely paraphrased by Pollack (2001): “if the water clings to the matrix, column weight is irrelevant: so long as there is enough adhesive force, a sufficiently long tube could deliver water to the moon.” Clearly, column weight does not necessarily limit tree height. Indeed, pectin-like fibrillar cell wall deposits have been detected in xylem fibers of poplar by Arend et al. (2008).

Once more, what is the significance of the Scholander balancing pressure P_b_?

A plausible function of pectin-like mucilage on the surface of leaves as well as in epistomatal caves and xylem vessels emerged from a study on several tree species including high-salinity-tolerant ones (D. Zimmermann et al. 2007). Height-dependent profiles of P_b_ have been measured on 7- up to 60-m-tall trees of *Eucalyptus pilularis* from the coastal region of NSW, Australia, and two 35-m-tall poplar trees of *Salix nigra* from the Tauber river valley near Würzburg. On *Eucalyptus*, 260 P_b_ values were collected between 1 to 57 m height, on poplar 253 P_b_ values between 1 to 25 m height. The P_b_ values ranged between 0 and 2.5 MPa, and significantly correlate with the ambient relative humidity, rather than with tree height! Concomitant xylem sap extraction analyses and ^1^H-NMR imaging of twigs, as well as microscopy and pectin staining by Alcian blue revealed that the mucilage formation indicates a local response of the canopy to water stress, for instance, due to sun exposure or drought. It should be noted that the conclusive outcome of this study relied upon cooperation of nine laboratories. The inferred role of pectic substances in plant water relations is emphasized by the ubiquity of epistomatal mucilage plugs, that is, in 67 gymnosperm and angiosperm species so far (Westhoff et al. 2009b).

After all, this study again demonstrates that the Scholander bomb is no reliable tool to support the C-T theory. However, if calibrated by the turgor pressure probe, it might help to monitor the plant water status (see below).

## Emboly repair by water secretion

A functional segmentation of the xylem conduit includes cavitation and emboly formation. A straightforward mechanism of xylem refilling would be water secretion from adjacent parenchyma cells, that is, beyond the equilibrium set by hydraulic coupling suggested already by Renner (1911). Hithertoo, active water secretion contradicted to an axiom of general physiology that water strictly moves thermodynamically downhill, possibly including aquaporins to facilitate transmembrane water permeation. In the recent years, however, convincing evidence emerged for uphill water transport by strict coupling to energized ion cotransport (Zeuthen 2010). In epithelial membranes, a cation-chloride-cotransporter couples one K^+^/Cl^-^ with up to 500 H_2_O molecules. Members of this CCC-transporter family have been found also in plants, namely *Arabidopsis*, *Oryza*, and *Grapevine* (Colmenero-Flores et al. 2007; Kong et al. 2011; Henderson et al. 2015). Preferential expression occurs in the xylem/symplast boundary of the vascular tissue of root and shoot.

**Fig. 1:**
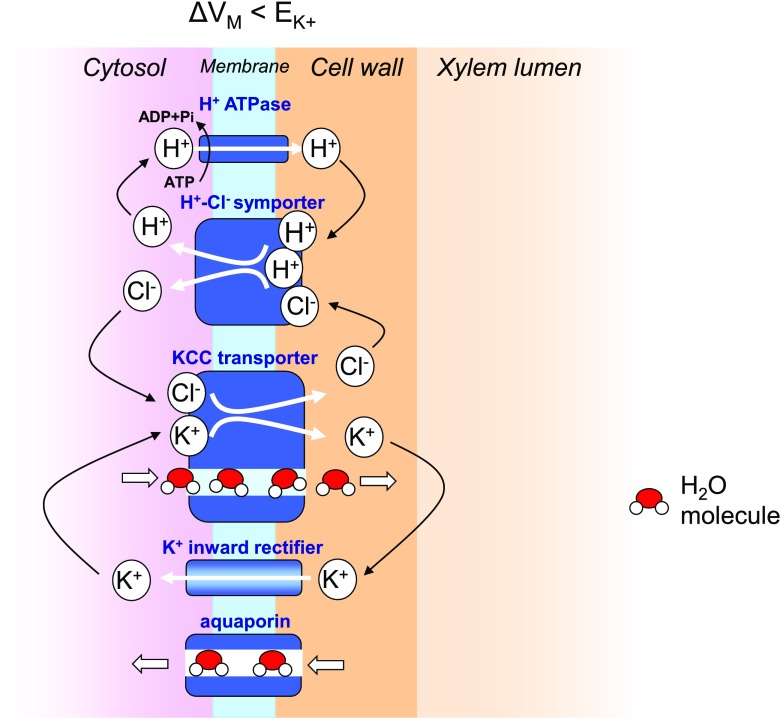
Emboly repair through water secretion to the xylem lumen by means of a hypothetical K^+^Cl^−^-cotransporter in the plasma membrane of xylem parenchyma cells. See text. From Wegner (2014a) with kind permission of Oxford University Press

Figure 1 illustrates how emboly repair could be facilitated by water secretion from the xylem parenchyma cells to the xylem conduit by means of a cotransporter proposed by Wegner (2014a). The cotransporter is energized by the plasma membrane H^+^ ATPase which recycles the Cl^−^ ions through a H^+^–Cl^−^ symporter and the K^+^ ions through a K^+^ channel responding to the membrane potential ΔV_m_.

Figure 1 also includes the known water channel aquaporin to indicate the regulatory aspect of passive transmembrane water flow.

Of course, this proposal is a genuine feature of both Otto Renner’s postulate and the functional xylem segmentation according to the watergate multi-force model of Zimmermann et al. (2004).

## Perspectives

Land plants evidently have learned to cope with water deficits by developing an amazing diversity of mechanisms imposed by the second law of thermodynamics. Experimental elucidation plausibly requires a likewise diverse array of tools. A detailed concept extending from the molecular level to the whole plant in the field is outlined by Wegner (2014b, 2015). Interesting reading also is a recently published physicist’s view. Inspection of the enormous bibliography led Brown (2013) to conclude that the C-T-theory “is not the whole story.” He notes, for instance, the already well-founded skepticism of Copeland (1902).

### Plant water status going online

A non-invasive leaf patch clamp pressure probe (LPCP) for a continuous monitoring of the plant water status in the field has been developed by Dirk and Ulrich Zimmermann (2008). The output pressure signal *P*_*p*_ may be calibrated by the turgor pressure probe, so the LPCP records temporal and spatial dynamics of the leaf water status of trees with high accuracy. The LPCP operates online featuring a real-time remote control by telemetry and data transfer to the internet, especially in the water management of crop plants (Rüger et al. 2010). Therefore, this instrument is a promising tool to gain profound insights into the physiology and ecology of land plant (Zimmermann et al. 2013).

Interestingly, concomitant recordings of the turgor pressure *P*_*c*_, the LPCP output *P*_*p*_, and the Scholander balancing pressure *P*_*b*_ revealed that *P*_*p*_ and *P*_*b*_ are inversely correlated to the turgor pressure (see figures 4 and 5 in Rüger et al. 2010). Thus, within the bounds of the rather limited accuracy of *P*_*b*_, both tools measure relative changes in turgor pressure.

## Concluding remarks

### The Max Planck paradigm

“A new great scientific idea tends to succeed not by eventually convincing the opponents, rather by prevailing because the opponents eventually die out and the next generation grows up with the new idea” (Planck 1933). The new idea was the thermodynamics of irreversible processes introduced by Rudolf Clausius in 1876, which had been the topic of Planck’s Doctoral Dissertation, 1879 in München. Planck complained “…that in the physics community the impact of the thesis was equal to zero. None of my academic teachers – I know it from talking to them – realized its significance …. Helmholtz seemingly had not read it at all, Kirchhoff explicitly refused it” (Planck 1943).

However, Helmholtz soon realized the fundamental significance of the new idea proposed by R. Clausius and J. Willard Gibbs, and even wrote a letter of acknowledgments to Gibbs. After all, it is a nice incidence that the second law of thermodynamics plays a role both in the academic career of Max Planck as well as in the history of the C-T theory.

### In the wake of Otto Renner (1883–1960)

Otto Renner (1883–1960) was an eminent scientist. His profound research on the cytogenetics of *Oenothera* led the plant geneticists Friedrich Oehlkers and Fritz von Wettstein to give credit to Renner that the theory of plastid DNA inheritance eventually was established (Hagemann and Eichhorn 2012). Secondly, in 1907, after a stay of some months with Wilhelm Pfeffer in Leipzig, Renner turned to the controversial field of water ascent. A priori, he favored the C-T mechanism, because it would be consistent with the capillary force-based imbibition theory of Julius Sachs (Renner 1910). On the other hand, Renner shared the reasoning of Wilhelm Pfeffer regarding the osmotic role of the parenchyma tissues and also adopted Pfeffer’s standards of physicochemical theory and experiment (Renner 1911, 1915). Therefore, although he was given credit for his experimental support of the C-T theory, Renner nevertheless remained skeptic throughout his life, missing conclusive evidence for the imaginary continuous water columns in the xylem conduit.

In closing, I would like to add that Otto Renner was an eminent scientist and an honest man as well. The likewise eminent scientist Erwin Bünning had spent years with Otto Renner at the Botany Institute in Jena. In his fascinating recollections, Bünning (1977) wrote: “How much good reason we had to trust and admire our professor (Otto Renner) became clear in 1933…in May 1933, Renner risked a very strong public attack against the Nazis. In the introduction to a seminar he described the great role of Jewish scientists in Germany, and defended Leo Brauner, a Jew who at that time was already forbidden to enter the institute.”

Otto Renner left Jena in 1948 to accept the Botany chair in München, succeeded by Leo Brauner in 1955.
